# FISH and tips: a large scale analysis of automated versus manual scoring for sperm aneuploidy detection

**DOI:** 10.1186/2051-4190-23-13

**Published:** 2013-12-01

**Authors:** Guillaume Martinez, Pierre Gillois, Marine Le Mitouard, Rémy Borye, Camille Esquerré-Lamare, Véronique Satre, Louis Bujan, Sylviane Hennebicq

**Affiliations:** Genetic and procreation Department, CS 10217, CHU de Grenoble, Laboratoire d’aide à la procréation – CECOS, Grenoble, Cedex 9, 38043 France; Joseph Fourier University, Grenoble, F-38000 France; Laboratoire AGIM, CNRS FRE3405, “Equipe Andrologie Génétique et Cycle cellulaire”, La Tronche, F-38700 France; ThEMAS TIMC-IMAG, UMR CNRS 5525, Joseph Fourier University, Public Health Pole, University Hospital of Grenoble, BP217, Grenoble, Cedex 9, 38043 France; CECOS and Toulouse University, UPS, Groupe de recherche en fertilité humaine (EA3694, Human Fertility Research Group), Hôpital Paule de Viguier, University Hospital of Toulouse, Toulouse, France; Genetic and Procreation Department, CS10217, CHU de Grenoble, Génétique chromosomique, Grenoble, Cedex 9, 38043 France

**Keywords:** Aneuploidy, Automation, Laboratory, Infertility, In Situ Hybridization, Fluorescence, Spermatozoa

## Abstract

**Background:**

Approximately 1% of the spermatozoa found in ejaculate of healthy men are aneuploid and this rate increases in the population of subfertile and infertile men. Moreover, fertilization with these aneuploid sperm can lead to impaired embryo development. Fluorescent In Situ Hybridization (FISH) is the common cytogenetic tool used for aneuploidy screening on sperm. However, it is a time-consuming technique and cytogenetic or in vitro fertilization laboratories cannot routinely use it and face the increasing demand of such analyses before Assisted Reproductive Techniques (ART). As automation can be a clue for routine practice, this study compares manual and automated scoring of sperm aneuploidy rates using a Metafer Metasystems® device. The results obtained also contribute to global data about FISH on sperm cells.

**Methods:**

We recruited 100 men addressed for sperm cryopreservation. They all signed an informed consent to participate in the study. 29 men were donors or consulted before vasectomy (control group) and 71 were suffering of Hodgkin’s disease or non Hodgkin lymphoma (patient group). One semen sample was collected for each patient, analyzed according to WHO criteria and prepared for a triple-color FISH using centromeric probes for chromosomes 18, X and Y. Automated scoring was performed using a Metafer Metasystems® device.

**Results:**

507,019 cells were scored. We found a strong concordance between the automated and the manual reading (d < 0.01 in Bland-Altman test). We also did not find a statistically significant difference between the automated and the manual reading using Wilcoxon test for total aneuploidy rate (p = 0.06), sex chromosomes disomy (p = 0.33), chromosome 18 disomy (p = 0.39) and diploidy (p = 0.21). Cumulative rate of total aneuploidy was 0.78% ± 0.212% for patient group and 0.54% ± 0.15 for control group and among this, sex chromosome XY disomy rate was of 0.54% for patient group and 0.27% for control group.

**Conclusion:**

This study validates the automated reading for FISH on sperm with a Metafer Metasystems® device and allows its use in a laboratory routine.

## Abstract (fr)

**Contexte:**

Le taux d’aneuploïdies spermatiques est d’environ 1% pour les hommes fertiles et augmente notablement dans la population des individus infertiles. L’obtention d’une fécondation à partir de ces spermatozoïdes aneuploïdes peut entrainer des anomalies de développement embryonnaire. L’évaluation des taux d’aneuploïdies est actuellement possible de façon simple par hybridation in situ fluorescente, toutefois la lecture manuelle des signaux obtenus est longue et fastidieuse. Les techniques de lecture automatisée des spots fluorescents se sont développées ces dernières années et sont actuellement de plus en plus utilisées en cytogénétique de routine. L’application de ces techniques aux spermatozoïdes permettrait donc une évaluation plus régulière des aneuploïdies spermatiques en infertilité. Nous présentons une étude comparée de la lecture manuelle et de la lecture automatisée en système Metafer Metasystem® sur un échantillon important de témoins fertiles et de patients à caryotype normal.

**Méthode:**

100 hommes consultant pour congélation de spermatozoïdes ont été inclus dans l’étude après information et recueil de leur consentement écrit. Cet échantillon était divisé en 29 donneurs fertiles ou patients consultant avant vasectomie et 71 patients consultant dans le cadre d’une maladie de Hodgkin ou d’un lymphome non hodgkinien. Un recueil a été réservé pour l’étude, les paramètres spermatiques ont été analysés selon les recommandations de l’OMS. Ensuite les taux d’aneuploïdies ont été évalués par FISH pour les chromosomes X, Y et 18 en système à trois couleurs utilisant des sondes centromériques.

**Résultats:**

507,019 spermatozoïdes ont été analysés. La concordance entre les deux modes de lecture est forte (d < 0.01 in Bland-Altman test) et aucune différence n’a été observée entre la lecture automatique et manuelle au test de Wilcoxon (p > 0,05) pour le taux global d’aneuploïdies, les disomies des chromosomes sexuels ou du chromosome 18 et les diploïdies. Le taux global d’aneuploïdies était de 0.78% ± 0.212% pour les patients et 0.54% ± 0.15 pour les témoins fertiles et le taux de disomies XY était de 0.54% chez les patients et 0.27% chez les témoins.

**Conclusion:**

Les données présentées dans ce travail permettent de valider une utilisation du lecteur automatisé de spots Metafer Metasystems® pour l’analyse chromosomique des spermatozoïdes en routine.

## Background

Infertility is defined as the inability to conceive after one year of unprotected regular coitus. It concerns one in six couples [1]. The origin of the infertility remains undefined in 15% of the cases. Male factors are involved in approximately half of the other 85% (associated or not with a female factor) [2, 3]. Many factors can influence male fertility and genetic abnormalities are involved in about 15% of the cases [4]. These male genetic defects are currently divided in single gene disorders (CFTR gene mutations for instance), Y chromosome deletions (AZF regions), structural and/or numerical chromosome abnormalities. Concerning chromosomal abnormalities origin, approximately 1% of the spermatozoa found in the ejaculates of healthy men are aneuploid [5]. This rate increases in the population of subfertile and infertile men. The development of intracytoplasmic sperm injection (ICSI) has widened the range of male infertility treated by intra-couple Assisted Reproductive Techniques (ART) but raised the problem of an increased risk of transmitting an aneuploidy to the offspring. Most of the aneuploid concepti lead to miscarriage but some of them are viable. About 0.2 of live births are aneuploid conceptions [6] and most of them are 13, 18 or 21 trisomies or dysgonosomy. Sex chromosomes are more prone to non-disjunction than autosomes [7]. Autosomal trisomies frequently have a maternal origin (95% of chromosome 21 trisomy and 93% of chromosome 18 trisomy 18), while sex chromosomal aneuploidies have a paternal one (100% of 47,XYY, 70-80% of 45,X and 50% of 47,XXY) [8]. Among the infertile men, sperm aneuploidy rates are higher than, in the fertile men. This increase has been described for all the chromosomes, but the most elevated rates are observed for chromosome X, Y, 21 and 22 [9, 10].

The Fluorescent In Situ Hybridization (FISH), using locus specific fluorescent probes, is a common cytogenetic tool employed for chromosome enumeration. Recent studies have supported the fact that FISH for aneuploidy screening on sperm cells should be incorporated as a routine prognostic test before a first ICSI attempt [11, 12]. Therefore, several thousands of spermatozoa are usually analyzed in order to be able to detect statistically significant differences between patients and controls even if they are low. This procedure, when performed manually by a trained technician is highly time consuming. Tempest and collaborators demonstrated that no difference is observed in aneuploidy rates when scoring 1000 cells or 5000 cells [13]. We evaluated several years ago, that adhering to strict scoring criteria and depending on the cell density on the slides, 3 to 15 hours are needed to enumerate the chromosomal content of 5.000 cells for a set of three probes. Even if the number of analyzed cells is lowered to 1000, when analyzing infertile patients, the time consumed remains high, since cell density on the slides is usually low. A good way to face the increasing demand of this time-consuming technique could be automation. Automated systems are already used in scoring genetic anomalies in different cells with a strong concordance between manual and automated screening results [14–16]. To our knowledge, four recent prospective pilot studies worked on the comparison between manual and automated approaches on sperm FISH [17–20]. They all agreed that the automated counting for FISH on sperm is a useful evolution but requires further validation on the different systems.

This study evaluates the reliability of the analytical method for detection of sperm aneuploidy in 100 samples by comparing the rates obtained after automatic reading with a METAFER Metasystems® device to manual reading. It also adds a contribution to global data about FISH on sperm cells.

## Materials and methods

### Patients

Between 2003 and 2008, a total of 100 men who were adressed for sperm donation or cryopreservation before vasectomy (controls, n = 29) or cryopreservation before gonadotoxic treatment for lymphoma (patients, n = 71), were recruited to perform sperm aneuploidy analysis in a multicenter protocol survey of chemotherapy and radiotherapy gonadotoxic effects on sperm (GAMATOX project: PHRC 02011601). The study was approved by the Ethics Committee of the University Hospital of Toulouse (Comité de Protection des Personnes Sud-Ouest et Outre Mer). All patients signed an informed consent to participate in the study.

### Sperm analysis

Semen samples were collected in a sterile container after masturbation. Liquefaction was obtained after 30 min at 37°C. Sperm concentration, progressive motility and round cells concentration were determined according to WHO criteria [21]. As aneuploidy rates are not significantly different in fresh and frozen sperm [22, 23], all the samples were frozen in order to perform the evaluation of aneuploidy rates altogether at one time. Therefore, after a 1/0.7 dilution in Sperm Freeze® (FertiPro NV, Belgium), the remaining sperm was frozen in high security straws (CryBioSystem, IMV Technologies, USA) using an automated procedure and stored in liquid nitrogen. All the samples were then included in the Research Biobank GERMETHEQUE after a standardized procedure of anonymization.

### FISH procedure

For each patient or control, one straw was thawed at 37°C for 10 minutes. Samples were washed twice with 5 ml of PBS 1X and fixed in a methanol/acetic acid (3:1,v/v) solution. Cells were spread on Superfrost© slides and air dried at room temperature. Sperm head decondensation was performed in NaOH 1 M solution, followed by two washes in 2X SSC and dehydration in a 70, 90% and pure ethanol solution. Samples were then hybridized with Abbott© Vysis centromeric probes (Abbot Laboratories, USA) CEP 18 (18p11.1-q11.1, D18Z1, SpectrumAqua), CEP X (Xp11.1-q11.1, DXZ1, SpectrumGreen) and CEP Y (Yp11.1-q11.1, DYZ3, SpectrumOrange), according to the Vysis probes protocol in a HYBrite® system (Abbot Laboratories, USA). Sperm nuclei were counterstained in a 0,5 μg/ml Hoechst solution for 3 minutes, washed in PBS 1X for 3 minutes and mounted with antifade.

### Aneuploidy scoring

Two trained technicians blind scored the slides on a Nikon Eclipse 80i epifluorescence microscope. Chromosomal aneuploidies were analyzed simultaneously for chromosomes 18, X and Y with Aqua, FITC and Spectrum Orange filters. In both situations, strict criteria [19] were used. Only clearly defined sperm nuclei with a flagella and containing at least one spot were analyzed and two signals of the same color were considered as different if they were the same size and were separated at least by a spot diameter. Since, the whole research protocol had been designed before data of Tempest and coll [13] were published, approximately 5000 cells were counted per sample, one half automatically, the other half manually. We did not modify the design because it also allowed us to detect statistical differences in each group (more than 1000 in the manual and in the automated group, and also between the two operators in the manual group). As it cannot be differenciated between nullosomic cell or hybridization defect, and most mososomic concepti are not viable, only disomic or diploid cells were considered. Each sperm cell was identified as normal (18,X or 18,Y), disomic for sex chromosomes (18,X,Y, 18,X,X and 18,Y,Y), disomic for chromosome 18 (18,18,X and 18,18,Y) or diploid (18,18,X,Y, or 18,18,X,X 18,18,Y,Y). All the combinations of spots counted in the cells during the automatic procedure were manually verified (using the strict criteria listed above) in the galleries of images provided by the machine. Each observer who performed either the manual reading or the automated reading and verification procedure evaluated the duration of the procedure.

### Data analysis

Data were treated with R software (version number 2.14.1). Sperm quantitatives parameters observed for controls, Hodgkin (H) patients and non-hodgkin lymphoma (NHL) patients were compared using the Student-t test. We used the Bland-Altman test to evaluate the concordance between the automated screening and the manual reading [24]. The variations between the two types of reading and inter-operator differences were analyzed using the Wilcoxon test for paired data. The Wilcoxon test was used to determine if a difference exists between the two types of reading for total aneuploidy, sex chromosome or chromosome 18 disomy and diploidy. We also used the Pearson’s product–moment correlation test to evaluate the correlation degree between the two types of reading.

## Results

Sperm analysis of our population of patients and controls is presented in Table 1 and revealed a mean sperm concentration in a normal range of 71.1 millions/ml for the controls and 58.6 millions/ml for the patients. When considering Hodgkin-s disease and NHL, mean sperm count was also normal of 57.8 and 60.9 millions/ml. No statistical difference was observed between the groups for this parameter (Controls vs Patients : p-value = 0.29 ; controls vs Hodgkin: p-value = 0.29 ; controls vs NHL: p-value = 0.58 ; Hodgkin vs NHL: p-value = 0.85). Sperm motility was also in a normal range in all the groups and so was progressive motility too. The values were respectively of 44.3 and 43.9% for controls and patients and 40.8 and 53.0% for Hodgkin and NHL patients. No statistical difference was observed among the groups (controls vs Hodgkin: p-value = 0.18 ; controls vs NHL: p-value = 0.06, NS ; Hodgkin vs NHL: p-value = 0.30). Sperm vitality was normal in all the groups (controls: 70.9%, Patients: 60.9%, Hodgkin: 61.6%, NHL: 58.7%) but a significant difference was observed between controls and both types of patients (controls vs Hodgkin: p-value = 0.01 ; controls vs NHL: p-value = 0.02) while not between Hodgkin and NHL groups (p-value = 0.56).

**Table 1:** **Sperm analysis**

		Controls	Patients		
Sperm analysis		n = 29	Total	Hodgkin	NHL
			n = 71	n = 53	n = 18
**Sperm concentration (10** ^**6**^ **/ml)**	**mean**	**71.1**	**58.62**	**57.85**	**60.89**
	*SEM*	*46.74*	*38.27*	*36.43*	*43.70*
**Progressive motility**	**mean**	**44.34**	**43.92**	**40.83**	**53.00**
	*SEM*	*7.27*	*10.20*	*10.20*	*10.44*
**Sperm viability**	**mean**	**70.89**	60.90*	61.65*	58.71*
	*SEM*	*10.75*	*12.86*	*12.86*	*13.96*

Mean value with standard error to the mean (SEM) of sperm parameters for control and patient groups, and for Hodgkin disease and non-Hodgkin lymphoma (NHL) groups (*p < 0.05 versus controls).

For aneuploidy scoring, an average of 4,970 sperm was analyzed per sample, half manually and half with the automated device, for a total of 507,019 cells: 256,539 manually and 250,480 automatically.

**Figure 1:**
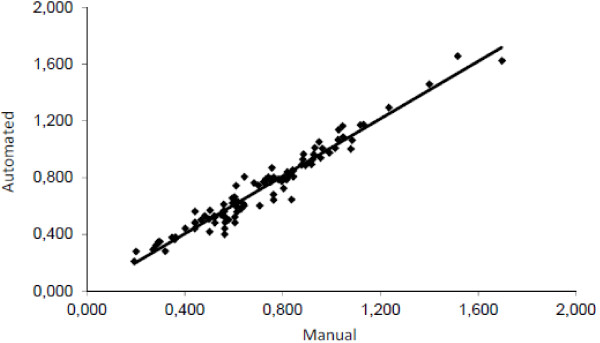
**Pearson’s correlation rate of total aneuploidy rate (R** ^**2**^ **= 0,9489).**

Using Wilcoxon test, our results did not show a statistically significant difference between the automatic and manual readings for the total aneuploidy rate (p-value = 0.06), the disomies of sex chromosomes (p-value = 0.33), the disomies of chromosome 18 (p-value = 0.39) and the diploidy (p-value = 0.21). In addition, no statistically significant inter-operator difference was found (p-value = 0.34). We also obtained a good correlation between both readings (Figure 1) for total aneuploidy rate (R^2^ = 0.96, p-value < 0.01) using the Pearson’s correlation test.

**Figure 2:**
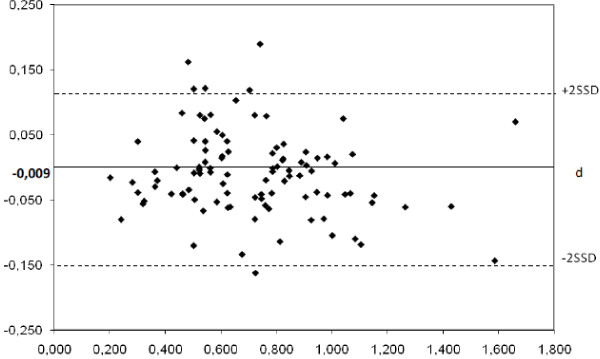
**Evaluation of non-inferiority between manual and automated scoring for total aneuploidy rate by Bland-Altman plot.** Each of the paired measures is represented by assigning the average of the two rates in abscissa and the difference between the two measurements in ordinate (d = -0.009; 2SSD = 0,127).

We found a strong concordance (Figure 2) between the automated screening (tested method) and the manual reading (reference method) with the Bland Altman test (2ssd = 0.13, d = -0.009, NS) for total aneuploidy rates.

We also analyzed the time required to analyze the slides in both methods. Classifying 2500 cells on good quality slides lasted a mean time of 3 hours manually vs one hour by the machine followed by ¾ hours for the operator to check the images in the gallery. When analyzing bad quality slides, the required average time was 12 hours manually vs 4 hours by the machine followed by 3 hours of re-analysis by the operator. We also tried different techniques of spreading or decondensation without improvement of bad quality slides. The average gain provided by the machine was 75% of operator time when considering that the machine was reading the slides during the night.

Considering all samples, we obtained a total euploidy rate of 99.29%, (Table 2) and thus a cumulative rate of total aneuploidy rate (sex chromosome, chromosome 18 and diploidy) of 0.72%. The sex chromosome disomies were the most frequently observed and represented 0.5% of the sperm cells analyzed (0.36% 18,X,Y, 0.07% 18,X,X, 0.07% 18,Y,Y). The disomy rate of chromosome 18 was 0.12% (0.06% 18,18,X, 0.06% 18,18,Y) and the diploidy rate was 0.1% (0.06% 18,X,Y, 0.02% 18,X,X; 0.02% 18,Y,Y).

**Table 2:** **Comparison of manual and automated scoring for all the chromosomes analyzed**

	Manual	Automated	Total
18,X	50.16	49.60	49.88
18,Y	49.13	49.68	49.41
**Total euploidy rate**	**99.29**	**99.28**	**99.29**
18,X,Y	0.36	0.36	0.36
18,X,X	0.07	0.07	0.07
18,Y,Y	0.07	0.06	0.07
**Total sex disomy rate**	**0.50**	**0.49**	**0.50**
18,18,X	0.06	0.06	0.06
18,18,Y	0.06	0.06	0.06
**Total 18 disomy rate**	**0.12**	**0.12**	**0.12**
18,18,X,Y	0.06	0.06	0.06
18,18,X,X	0.02	0.02	0.02
18,18,Y,Y	0.02	0.02	0.02
**Total diploidy rate**	**0.10**	**0.10**	**0.10**
**Total aneuploidy rate**	**0.72**	**0.71**	**0.72**

The table presents the detailed aneuploidy rates (%) for all the chromosomes analyzed by manual and automated scoring for all the patients and controls.

Total aneuploidy rate was statistically different between controls and both patient groups (Table 3), (controls vs Hodgkin: t = 4.69, df = 68.13, p-value < 0.01, S ; controls vs NHL: t = 2.33, df = 22.45, p-value = 0.03, S) and no difference between Hodgkin and NLH groups (t = 0.01, df = 21.58, p-value = 0.99, NS) was observed. The mean rate was of 0.56% for the controls, 0,78% for the patients and among the patients of 0.78% for the Hodgkin and 0.77% for the NHL groups.

**Table 3:** **Comparison of total aneuploidy rates among the control and the patient groups**

		Controls	Patients		
Total aneuploidy rate		n = 29	Total	Hodgkin	NHL
			n = 71	n = 53	n = 18
**Manual**	**mean**	**0.54**	0.78*	0.78*	0.76*
	*SEM*	*0.15*	*0.21*	*0.18*	*0.28*
**Automated**	**mean**	**0.57**	0.78*	0.78*	0.77*
	*SEM*	*0.15*	*0.23*	*0.21*	*0.29*
**Total**	**mean**	**0.56**	0.78*	0.78*	0.77*
	*SEM*	*0.15*	*0.22*	*0.19*	*0.29*

The table presents the mean value with standard error to the mean (SEM) of total aneuploidy rate for control and patient groups, and for Hodgkin disease and non-Hodgkin lymphoma (NHL) groups. (*p < 0.05 versus controls).

Despite the strict criteria used in scoring, which excluded most of the round cells from scoring, we also tested our data for the possible scoring of round cells. According to meiotic data, round cells could be diploid cells (premeiotic germ cells or somatic cells) or haploid post meiotic cells. In order to precise if diploid cells could be round cells, we looked for a correlation between the rates of diploid cells and the concentration of round cells. Wilcoxon test for paired data applied to these parameters revealed a statistical difference among these (p-value < 0.01).

## Discussion

Concerning aneuploidy scoring, the automated results are consistent with the manual ones and aneuploidy rates obtained by both methods are comparable for all our values. These results are in accordance with recent publications on the subject, using either a Metafer [20] or other systems (Spot AX system [18, 19] or BioImage and Atto Imaging Vision software [17]). But the largest study published so far included only 24 samples [19] on a Spot AX system and our study is now the largest one.

A second point to be discussed is the ability of the machine to classify the spots in the cells without any error. Since the automated method was in evaluation, manual verification of the gallery was done systematically, in order to avoid any misclassification done by the software. For good quality slides, the rate of misclassified cells remained low. For bad quality slides, the increase of 3 to 4 fold of the time needed to manually reclassify the cells attested of a higher rate of misclassification by the machine. Thus, in our hands, whatever the parameters of automated reading we used, the galleries of images needed to be verified to correct misreadings or eliminate cells which did not fit the strict criteria mentioned previously. Despite this quite negative aspect, the use of the machine was still advantageous since the time devoted to this verification step, was 3 fold shorter than the manual reading of the spots even for bad quality slides. Moreover, it was much easier and convenient for the technician to classify the galleries than read directly through the microscope. Our results are thus in accordance with other results published by Carrel and collaborators on the Metafer Metasystem® device [20, 25].

The system can thus automatically analyze a large number of samples per day making the sperm-FISH technique more accessible. In addition, the software allows data saving for a future use. Fading of the slides after automated reading is less important than in manual reading, allowing several readings of the same slide, which is usually quite difficult in manual reading. Moreover, relocation of the cells can be done in case of doubt on a result given by the controller, which is not possible when reading is manual.

Our study is also the first to obtain a correlation for diploidy rates between both evaluations. The studies using automated reading have obtained very heterogeneous results in diploidy analysis so far. This can be explained by the lack of statistical power in previous studies and also by the fact that diploid sperm may be difficult to identify. Indeed, the diploid sperm head is usually larger and rounder than a haploid one. Determining if the cell is a diploid sperm or a diploid round germ cell present in semen can thus be difficult. When using strict reading criteria, cells suspected as diploid must have only one flagella to prevent from analyzing overlapped sperms. The sperm head basis must be morphologically normal since a too strong decondensation modifies the sperm head morphology and prevents from a correct scoring. For the decondensation step, protocols differ among publications. The three main processes used are sodium hydroxide, DTT Tris–HCl or heating. In our experience, whatever the protocol used, it has no effect on subsequent results (unpublished data). From this first part of our study, we can conclude that the automated counting can be substituted to manual reading.

The second part of our study focused on the analysis of sperm parameters and aneuploidy rates for lymphoma patients vs controls. Results observed in the literature concerning sperm parameters of patients with lymphoma differ from one study to another. As O’flaherty et al. [26], we did not find statistical differences between controls and patients for sperm concentration and motility, but another study [27] shows a decrease of these parameters for lymphoma patients. These discrepancies can be explained by the following causes: patients recruited for these studies have different basal sperm parameters, different individual parameters like age or exposition to environmental toxics or different stages of the disease. Moreover, some had fever during the disease that may have impaired their spermatogenesis [28]. Our results only showed a significant decrease in viability for both patient groups but the mean viability remained in a normal range in all the groups.

Concerning the global data on sperm chromosomal abnormalities, the aneuploidy rates of our controls were quite consistent with the published results [7, 29]. This study provides for the first time data on sperm aneuploidy in a large cohort of lymphoma patients before gonadotoxic treatment. Several studies describe a non significant increase in aneuploidy rates for lymphoma patients [30–33]. In our study, the rates for our patients appear significantly higher than those observed for the controls. Our analysis of a large cohort of patients shows with a higher statistical power, the increase observed by others on smaller cohorts. We thus can clearly imply an impact of the disease itself on sperm aneuploidy rates, although the mechanism of this phenomenon is yet not understood. We tested if the increase may be related to higher aneuploidy rates for oligozoospermic patients in the lymphoma group. This was not the case, since no difference in aneuploidy rate was observed according to sperm count in the patient group (data not shown). More work has to be done to elucidate the impact of the pathology on aneuploidy rates.

## Conclusion

Validation of automated reading for sperm-FISH is a major technical breakthrough that allows us to consider routine use of this technique in case of couples consulting for infertility problems. To date, the sperm-FISH technique does not allow sperm selection for In-Vitro Fertilization with Intra Cytoplasmic Sperm Injection. However, it provides useful information as an indicator of failure rate. Beyond the use of automated spot counters and/or image analysis systems, it would be appropriate for the next technical evolution to perform these techniques by flow cytometry. The data acquisition would be faster and the number of analyzed events more consistent.

## Abbreviations

ART: Assisted reproductive techniques
DTT: Dithiotreitol
FISH: Fluorescent in situ hybridization
FITC: Fluerescein isothiocyanate
GAMATOX: Gamète male toxicité (toxicity to male germ cell)
HD: Hodgkin’s disease
ICSI: Intra cytoplasmic sperm injection
NHL: Non Hodgkin lymphoma
PHRC: Projet hospitalier de recherche clinique (clinical research trial)
WHO: World health organization.
